# Experience of a TelEmergency program in Colombia South America: descriptive observational study between 2019 and 2021

**DOI:** 10.1186/s12873-023-00842-6

**Published:** 2023-07-04

**Authors:** Carlos E. Vallejo-Bocanumen, Daniel Pérez-Martínez, Diana Carolina Quiceno-Salazar, Yésica Paola Mejía-Gonzalez, Juan F. García-Cano, Diana C. Martínez-Pérez

**Affiliations:** 1 Urgencies and Emergencies Research Group, Faculty of Medicine, University of Antioquia, Carrera 51D #62 – 29, Office MUA 302, 050010 Medellín, Colombia; 2 Digital Hospital, Faculty of Medicine, University of Antioquia, Medellín, Colombia

**Keywords:** Telemedicine ER, TelEmergency, Emergency Care, Colombia

## Abstract

**Introduction:**

Colombia has 50,912,429 inhabitants, but only 50–70% of the population can effectively access health care services. The emergency room (ER) is a main contributor to the in-hospital care system since up to half of the admissions come through it. Telemedicine has become a tool to facilitate effective access to health care services, improve the timeliness of care, reduce diagnostic variability, and reduce costs associated with health. The aim of this study is to describe the experience of a Distance Emergency Care Program through Telemedicine (TelEmergency) to improve specialist access for patients at the Emergency Room (ER) in low- and medium-level care hospitals in Colombia.

**Methods:**

An observational descriptive study of a cohort including 1,544 patients during the program’s first two years was conducted. Descriptive statistics were used to analyze the available data. The data are presented with summarized statistics of sociodemographic, clinical, and patient-care variables.

**Results:**

The study included a total of 1,544 patients, and the majority were adults between 60 and 79 years of age (*n* = 491, 32%). More than half were men (*n* = 832, 54%), and 68% (*n* = 1,057) belonged to the contributory health care regime. The service was requested from 346 municipalities, 70% (*n* = 1,076) from intermediate and rural settings. The most common diagnoses were related to COVID-19 (*n* = 356, 22%), respiratory diseases (*n* = 217, 14%), and cardiovascular diseases (*n* = 162, 10%). We observed 44% (*n* = 681) of local admissions either under observation (*n* = 53, 3%) or hospitalization (*n* = 380, 24%), limiting the need for hospital transfers.

Program operation data revealed that 50% (*n* = 799) of requests were answered within two hours by the medical staff. The initial diagnosis was modified in 7% (*n* = 119) of the patients after being evaluated by specialists at the TelEmergency program.

**Conclusions:**

This study shows the operational data collected during the first two years after the implementation of the TelEmergency program in Colombia, the first of its kind in the country. Its implementation offered specialized timely management of patients at the ER in low- and medium-level care hospitals, where there is no availability of specialized doctors.

**Supplementary Information:**

The online version contains supplementary material available at 10.1186/s12873-023-00842-6.

## Introduction

Colombia has 50,912,429 inhabitants, with 50.4% being females, and 76% of the total population resides in urban areas. Its population pyramid is regressive, which resembles the demographic distribution of high-income countries. Even though in 2020 the health care coverage was 98%, only 50–70% of the population could effectively access health care services [1]. Variables such as socioeconomic and educational level, the place of residence, the geographic location of the health care institutions, and some administrative barriers can explain the gap [2, 3]. The health care system is a mix of public and private for-profit insurers and health care providers. Health care providers are mostly organized in institutions, such as clinics and hospitals [4, 5].

Telemedicine is a medical-care modality offered through information and communication technologies (ICT); it emerged as an alternative to address the challenges of global health systems. In context, telemedicine has become a tool to facilitate effective access to health care services, improve the timeliness of care, reduce diagnostic variability, and reduce costs associated with health [6, 7].

Colombian Law 1419 of 2010 supports the health care system through ICT and has fostered the progress of telemedicine in the country [8]. The University of Antioquia Faculty of Medicine has created a service-delivery model based on telehealth, called Living Lab or Digital Hospital. Currently, it has the TelEmergency Program, which is the name given to the Telemedicine Program specifically for Emergency Medicine, created in 2019 [9].

The emergency room (ER) is a main contributor to the in-hospital care system since up to half of the hospital admissions come through it; as the main access door for individuals trying to access health care systems, overcrowding can occur, which negatively impacts the quality of care [10]. Hospital access through the ER makes it easier and faster to find medical attention, even if the patient has a low triage category [11]. This situation reflects the structural flow barriers in the system, specifically in outpatient care.

The aim of this study is to describe the experience of the TelEmergency Program of the Digital Hospital from the University of Antioquia Faculty of Medicine between 2019 and 2021 in Colombia.

## Methods

### Type of study

This was a descriptive, cross-sectional observational study based on the database and clinical records of patients treated at the University of Antioquia’s Digital Hospital’s TelEmergency Program between December 19, 2019, and June 15, 2021.

### Scenario and study population

The TelEmergency Program provides specialized telemedicine support through the expertise of an Emergency Medicine Physician (EMP) to General Practitioners (GPs) in low- and medium-level care hospitals in Colombia (Telexpertise model). The TelEmergency Program is activated by the insurer when they receive a GP request for a hospital transfer of a specific noncritical patient. These referrals can be triggered due to the lack of specific resources, the need for diagnosis procedures, therapeutic support, or specialized assessment or care that cannot be done at the site of origin of the case. The core program’s objectives are to improve the quality of care, optimize the use of resources, avoid unnecessary hospital transfers, and uncrowd the ER [9].

Initially, GPs in low- and medium-level care hospitals who considered the patient would benefit from the TelEmergency Program created the “Request for Care” with the insurer, which would subsequently contact the digital hospital. The latter is based in Medellín, Colombia, and is on duty twenty-four hours a day, seven days a week.

The procedure includes a prehospital care technician receiving the notification, clinical records, images if available, and a "Request for Care" sent by the insurer. The next step is to verify the information, clinical documentation, and consent of the insurer, the patient, and the doctor before carrying out the care through the TelEmergency Program.

After the case and documents are cleared to follow through, the EMP on call is informed. From anywhere with an internet connection, they can access the platform, review the clinical records available, and – if needed – request additional information (text, images of the case, radiology results, laboratory tests, etc.). Through a phone call or video call, the EMP contacts the GPs in all cases to ask for updates on the patient's clinical status, ask for more information if needed and define the management and procedures to be followed, including hospitalization, referral, discharge, or reevaluation through telemedicine. The TelEmergency program flowchart can be found in the Supplementary Material*.*

The EMPs at the TelEmergency Program are clinically skilled, with more than five years of experience as attending physicians in the ER of low-, medium-, and high-level care hospitals all around the country, as well as nontechnical skills in communication with health care staff and knowledge about the TelEmergency strategy, its benefits, and limits for patient safety. Before beginning their duties, they are trained in the use of teleresources by the Information Technology (IT) team at the Digital Hospital.

The Digital Hospital keeps the medical records in a database based on the PostgreSQL ® system, which provides the possibility of verification and traceability of the entire process.

Colombia has 32 different insurers, but the population of this study consists of patients from a single insurer with more than 6,000,000 affiliates. Insurance premiums are paid either by the patient (Contributive Regime or CR, funded by salaried contributions) or subsidized by the government (Subsidized Regime or SR, partially or completely), depending on the individual´s ability to generate income and their social conditions. The population included were patients admitted to ERs or inpatients in low- and medium-level care hospitals across the country who required urgent hospital transfer for specialized assessment or treatment according to the criteria of the GPs. The observed period was between December 19, 2019, and June 15, 2021 (the first 18 months of the TelEmergency Program).

### Information collection and classification

The information was extracted from the database of and provided by the Data Analytics Department at the Digital Hospital. The data were shared through Excel files, and they were extracted from the patients’ medical records. All cases were included for analysis.

The variables included the patient's age, gender, health care affiliation regime, region of origin (municipality and department), initial and final diagnosis, medical conduct, and consult response time (the time between the consultation request and the digital hospital response).

### Statistical analysis

We performed a descriptive statistical analysis of the variables, with absolute values and percentages for categorical variables. For quantitative variables, we used the mean with standard deviation or median and interquartile range, depending on the data distribution.

“*Timeliness of Care”* was defined as the time span between the request made to the digital hospital and the EMP’s answer.

The variable “*Clinically Appropriated Request”* was defined as cases considered a good fit to be managed through the TelEmergency program by the EMP.

To determine the Program’s value-adding ability, the medical decisions were grouped as follows: 1. Hospital discharge; 2. Observation at the referral site; 3. Hospitalization in the primary unit; 4. Referral or transfer to another hospital; 5. Telemedicine consultation (with a specialist other than the EMP). These criteria were defined *a priori* by the TelEmergency Program.

For data processing, we used version 4.1.0 (R Foundation for Statistical Computing, Vienna, Austria) and RStudio (version 1.4.1717, R Studio Inc., Boston, U. S. A.) and Microsoft Excel^®^.

## Results

A total of 1,544 patients attended by the TelEmergency Program between December 2019 and June 2021 were identified. There were 1,493 patients (96.69%) who had only one consultation with the program; 49 patients (3.17%) received care on two occasions; one patient (0.06%) on three occasions; and one patient (0.06%) on four occasions, for a total of 1,598 requests. Of the 51 patients with more than one request, 25 (49.01%) of the requests were for the same diagnosis; 16 (31.37%) were for a similar one; and 13 (25.49%) were for a different diagnosis; of those, 12 (23.52%) subjects needed to be transferred.

The largest portion of the population was adults between 60 and 79 years of age (*n* = 491; 32%), and the average age was 54.2 (± 22.8) years. Most of the patients were men (*n* = 832; 54%). Two-thirds (*n* = 1,057; 68%) belonged to the CR; the rest were part of the SR. Participant characteristics are presented in Table 1.

**Table 1:** Sociodemographic variables of patients attended by the TelEmergency Program

Patient	1,544 (%)
Variables, n	
Age (years)	54.2 ± 22.8
0–5	29 (2)
6–11	16 (1)
12–18	53 (3)
19–39	351 (23)
40–59	383 (25)
60–79	491 (32)
80–105	221 (14)
Gender	
Female	712 (46)
Male	832 (54)
Insurer (Affiliation Regime)	
Contributory	1,057 (68)
Subsidized	487 (32)
Departments (States)	
Antioquia	595 (39)
Valle del Cauca	204 (13)
Cundinamarca	118 (8)
Cauca	115 (7)
Tolima	78 (5)
Others	434 (28)
Types of municipalities^d^	
Rural municipalities^a^	475 (31)
Intermediate municipalities^b^	601 (39)
Cities or agglomerations^c^	468 (30)

Unless otherwise indicated, the values express n (%) or mean ± Standard Deviation (SD)

^a^Rural municipalities have less than 25,000 inhabitants and present intermediate population densities (between 10 inhab/km2 and 100 inhab/km2)

^b^Intermediate municipalities have between 25,000 and 100,000 inhabitants in the municipal seat or have a high population density (more than 10 inhabitants/km2)

^c^Cities or agglomerations are municipalities with more than 100,000 inhabitants in their municipal seat or close to the previous ones, which allows behavior as agglomerated municipalities

^d^The classification of the type of municipality is taken from: National Planning Department, DNP, in Spanish, (2014). Definition of Rurality Categories (Thematic report for the Mission for the Transformation of the Countryside. Bogotá, D. C.)

Requests were registered from 346 municipalities from the 32 departments of Colombia, and 70% (*n* = 1,076) were generated from intermediate or rural municipalities. The types of municipalities (and definitions) and frequencies of requests to the TelEmergency program are shown in Table 1.

### Care and diagnosis

**Fig. 1:**
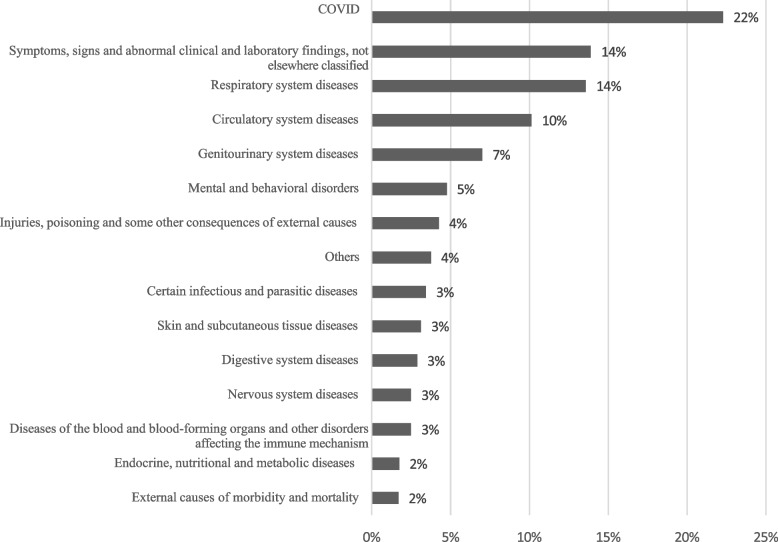
Distribution of diagnoses according to ICD-10

The diagnoses were grouped according to the International Classification of Diseases, Tenth Edition (ICD-10) [12], and their distribution is presented in Fig. 1. A total of 23% (*n* = 356) of the care was associated with the diagnosis of COVID-19 and belonged to a group of codes for special situations. This was followed in frequency by diagnosis groups in diseases of the respiratory system (*n* = 217; 14%); those of the circulatory system (*n* = 162; 10%); those of the genitourinary system (*n* = 112; 7%); and mental and behavioral disorders (*n* = 76; 5%). There was a total of 14% (*n* = 222) of the diagnoses grouped into symptoms, signs, and findings not classified elsewhere.

For all requests, the initial diagnosis was maintained in 92.55% (*n* = 1,479); in the other 7.44% (*n* = 119), there was a change in diagnosis, which could be due to a similar diagnosis due to some imprecision (*n* = 64; 4%) or due to a different diagnosis (*n* = 55; 3%).

The distribution of the final site of health care after consultation with the TelEmergency program is shown in Table 2. In one case, the care site was not described. There were no complications described in any of the patients.

**Table 2:** Final site of health care after the consultation to TelEmergency program

	*n* = 1544 (%)
Patients transfered	862 (55,82)
Medium-Complex Hospital	430 (27,84)
High-complex Hospital	397 (25,71)
Other health care facilities	27 (1,75)
Not transfered	681 (44,11)
Hospitalized	380 (24,61)
Discharged from TelEmergency program	192 (12,44)
Continued telemedicine management	56 (3,63)
Observation	53 (3,43)

*Timeliness of care* by the TelEmergency Program had a median of 2.06 h (interquartile range 1.24, 3.72); the fastest care was provided in 0.3 h.

## Discussion

This study describes the experience of a TelEmergency Program during the first two years after its implementation in Colombia South America. Its implementation offered specialized timely management of noncritical patients at the ER in low- and medium-level care hospitals, where there is no availability of specialized doctors, through a telexpertise model supported by ICT.

Before the implementation of the TelEmergency Program, all cases that required specialized medical evaluation were transferred to a hospital with a higher level of care. After the program´s implementation, 47% of the patients were kept or completed their attention at the hospital where the request originated. Studies show that the effect of telehealth on hospital transfers can go in different directions. In a systematic review, Toit et al. showed an increase in reported hospital transfers between 6.3 and 54.2% [13], while MacKinney et al. reported that up to 1.3% of transfers were avoided in a cohort of patients attended by a TelEmergency Program that served a large number of EDs in numerous states of the United States [14]. The result shown by the current study may mean more efficient use of resources in the health system.

Our data showed that 70% of the TelEmergency requests came from outside cities and metropolitan areas, distant from hospitals that can provide specialized care. This finding is important because geographic location is one of the greatest barriers to accessing health care services reported in the literature, especially in rural areas where the health care infrastructure is precarious, specifically in Latin America [15, 16].

In this study, we found that the median time for reaching specialized attention was 2.06 h. This possibility of having a specialized concept and monitoring can improve clinical outcomes [6]*.* Studies have shown that telehealth in the ER can improve timely access to specialist consultation and its positive impact on patient care [7, 17]. Many hospitals of low and medium complexity do not have the resources or enough volume of patients to justify hiring specialized medical staff, even an EMP; thus, this Telexpertise model tackles the lack of local resources for specialized assistance and timeliness of specialized medical evaluation that can expose patients to adverse outcomes [18, 19].

More than 1,500 ED inpatients were attended through this TelEmergency Program; all age groups received evaluation through it, and the majority were adults and males. Our findings related to sex coincide with the data published by Rahman et al. in Bangladesh, who found a profound gender disparity in telemedicine use during the COVID-19 pandemic, with fewer females than males. The reasons given by the authors include the difference in education levels and technical literacy in using online platforms for telehealth services [20].

However, a great number of studies have shown a positive effect of telemedicine in vulnerable populations, specifically women. As an example, Eberly et al. found that a higher proportion of women (4% higher than men) accessed primary care and specialized attention through telemedicine platforms during COVID-19 [21]. Additionally, Fischer et al. demonstrated that women tend to be more welcoming of telemedicine than men, but their study did not specify the modality used [22]. Another study conducted in Canada by Poder et al., related to a telehealth-based emergency trauma care program, reported that 51% of the population served were women [23]. The precise cause of this gap in the actual study is unknown and requires further research; however, some hypotheses could point to behavioral nuances or gender-related health care access barriers.

The diagnosis that topped the list of care was COVID-19, a fact that is explained by the overlap of the study period with that of the current pandemic, which, for months, has been challenging global health systems and – without exception – the Colombian system. There is already a review in the literature that shows that the distance-health-care modality in times of a pandemic has been implemented as a tool for evaluation and triage prior to care in the ER to increase the capacity of care in these services [24].

Regarding insurance, the number of patients belonging to the CR was considerably higher than those in the SR (close to a 2-to-1 ratio). This is related to the number of patients pertaining to each regime in the insurance carrier in 2019, where the people listed in the CR almost doubled those in the SR [25]. In the literature, some differences have been found depending on the insurance. Lattimore et al. found in a cohort of surgical outpatients who people enrolled in insurance programs other than those provided by the government are more likely to use telemedicine [26]. Karimi et al. found in a press release from the Office of Health Policy in the US that people with private health insurance tend to use telemedicine more often [27]. In Cali, Colombia, Escobar et al. reported their experience implementing a telemedicine program for outpatients, where charging copayments were the first cause of appointment cancellation from the patient side [28], an additional economic burden for the vulnerable population present in the SR. This finding requires further research, as it is important to establish the effects of insurance affiliation on telemedicine use in Colombia.

In Colombia, since 2019, telehealth and its modalities have been included in the Basic Health care Plans regardless of the type of insurance. However, the deployment of the programs depends on the agreements between insurers and service providers (clinics or hospitals), so the progress in the implementation of services is not homogeneous throughout the country [28, 29]. Other barriers that may arise when implementing emergency-telemedicine programs are funding, relationships among the interested parties, and trust from the medical staff, patients and their families with the remote-care modality [30].

Multiple TelEmergency strategies have been developed worldwide that can involve physicians and nursing personnel and can also be applied in the context of prehospital care, generating improvement in health care, patient flow across the health care system, and adding value in health management and administration (waiting times, avoidable transfers, communications, lower costs) [6, 7, 14]. In a systematic review, Ward et al. showed that telemedicine in ERs is an alternative with great potential to expand the use of services and address problems, such as overcrowding in urban centers and access in remote areas, which are undoubtedly some of the conflicts with which the Colombian health system must address [7].

To evaluate the impact of this TelEmergency Program in terms of quality of health care over different population groups, more information than is currently available and analytical studies are needed.

Information about costs was requested from the insurer carrier but was not made available for analysis.

## Conclusions

This study shows the operational data collected during the first two years after the implementation of the TelEmergency program in Colombia, the first of its kind in the country. Its implementation offered specialized timely management of patients at the ER in low- and medium-level care hospitals, where there is no availability of specialized doctors.

This TelEmergency Program can be considered representative in terms of accessibility and timeliness of care in noncritical patients who require evaluation by an EMP specialist, bearing in mind that providing medical attention in rural areas in Colombia is operationally difficult due to limited resources and staff shortages.

## Supplementary Information

**Additional file 1.**

## Data Availability

Datasets used and analyzed during the current study are available from the corresponding author upon reasonable request.

## Abbreviations

ER: Emergency Room
ICT: Information and communication technologies
CR: Contributive Regime
SR: Subsidized Regime
GP: General practitioner
EMP: Emergency Medicine Physician
IT: Information Technology
