# Supplementary material for: Experience of a TelEmergency program in Colombia South America: descriptive observational study between 2019 and 2021
Source: BMC Emerg Med. 2023 Jul 4;23:75. doi: 10.1186/s12873-023-00842-6 (PMC10321012; doi:10.1186/s12873-023-00842-6)
Supplement: Supplementary file 1 — Additional file 1. [file 12873_2023_842_MOESM1_ESM.pdf]

IMPLEMENTATION TELEMERGENY FRAMEWORK

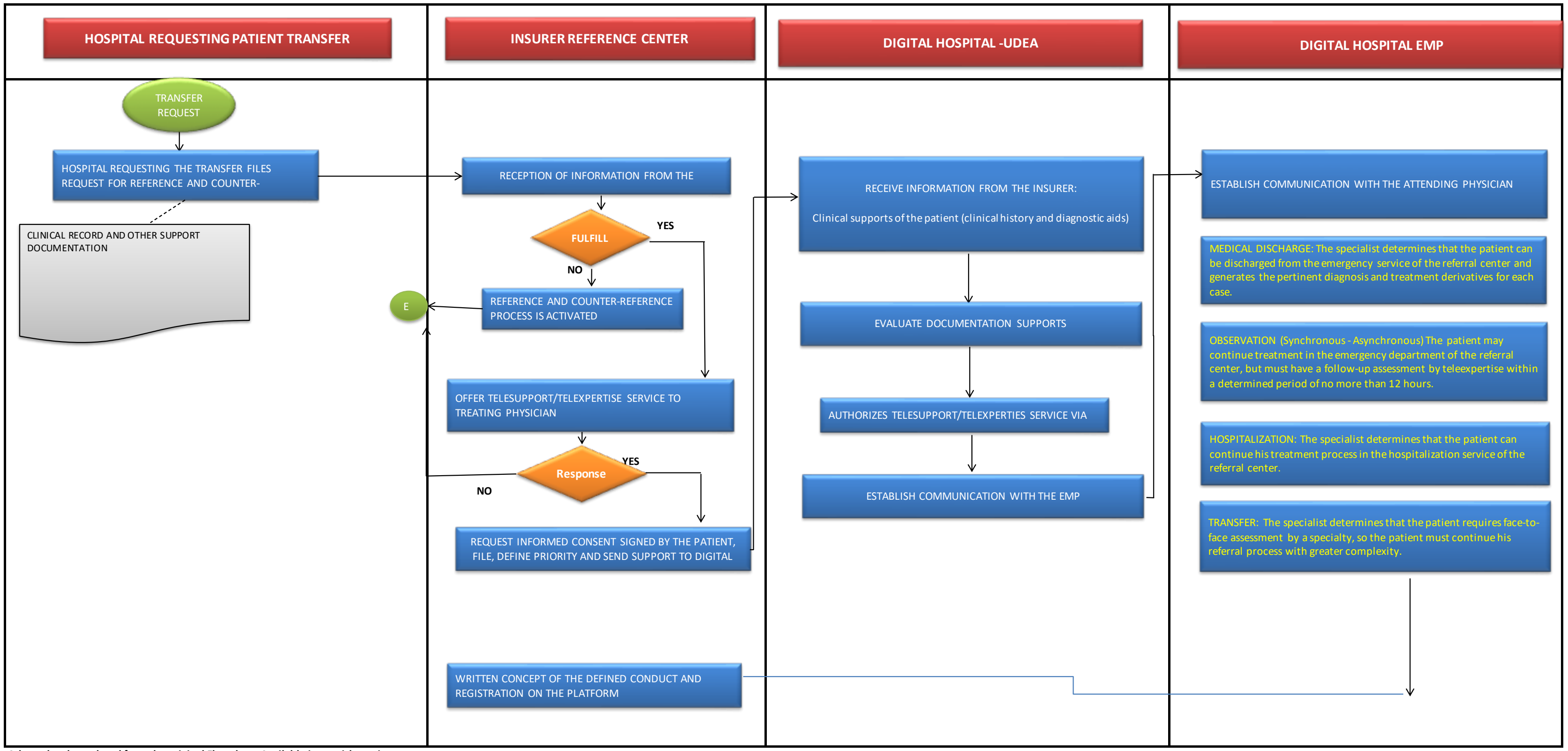

Adapted and translated from the original Flowchart. Available in spanish version.
